# Coordination of Zika Virus Infection and Viroplasm Organization by Microtubules and Microtubule-Organizing Centers

**DOI:** 10.3390/cells10123335

**Published:** 2021-11-27

**Authors:** Rebecca A. Buchwalter, Sarah C. Ogden, Sara B. York, Li Sun, Chunfeng Zheng, Christy Hammack, Yichen Cheng, Jieyan V. Chen, Allaura S. Cone, David G. Meckes, Hengli Tang, Timothy L. Megraw

**Affiliations:** 1 Department of Biomedical Sciences, Florida State University, Tallahassee, FL 32306, USA; rab07c@my.fsu.edu (R.A.B.); sara.york@med.fsu.edu (S.B.Y.); li.sun@med.fsu.edu (L.S.); chunfeng.zheng@med.fsu.edu (C.Z.); jieyanchen@gmail.com (J.V.C.); ams12s@my.fsu.edu (A.S.C.); david.meckes@med.fsu.edu (D.G.M.J.); 2 Department of Biological Science, Florida State University, Tallahassee, FL 32306, USA; sarcogden@gmail.com (S.C.O.); christyleigh.hammack@gmail.com (C.H.); ycheng4@fsu.edu (Y.C.); tang@bio.fsu.edu (H.T.)

**Keywords:** Zika virus, ZIKV, flavivirus, centrosome, centriole, microtubule-organizing center, MTOC, viroplasm, microtubule

## Abstract

Zika virus (ZIKV) became a global health concern in 2016 due to its links to congenital microcephaly and other birth defects. Flaviviruses, including ZIKV, reorganize the endoplasmic reticulum (ER) to form a viroplasm, a compartment where virus particles are assembled. Microtubules (MTs) and microtubule-organizing centers (MTOCs) coordinate structural and trafficking functions in the cell, and MTs also support replication of flaviviruses. Here we investigated the roles of MTs and the cell’s MTOCs on ZIKV viroplasm organization and virus production. We show that a toroidal-shaped viroplasm forms upon ZIKV infection, and MTs are organized at the viroplasm core and surrounding the viroplasm. We show that MTs are necessary for viroplasm organization and impact infectious virus production. In addition, the centrosome and the Golgi MTOC are closely associated with the viroplasm, and the centrosome coordinates the organization of the ZIKV viroplasm toroidal structure. Surprisingly, viroplasm formation and virus production are not significantly impaired when infected cells have no centrosomes and impaired Golgi MTOC, and we show that MTs are anchored to the viroplasm surface in these cells. We propose that the viroplasm is a site of MT organization, and the MTs organized at the viroplasm are sufficient for efficient virus production.

## 1. Introduction

First isolated from monkeys in the Zika forest of Uganda in 1947, Zika virus (ZIKV) is a mosquito-borne flavivirus, a genus of positive-sense single-stranded enveloped RNA viruses that include dengue (DENV), yellow fever, and West Nile viruses. Human transmission for ZIKV was rare until outbreaks occurred in Yap, Micronesia (2007), and French Polynesia (2013–2014). The 2015–2016 outbreak in the Americas, especially in Brazil, caused global health concern due to the associated microcephaly and additional birth defects collectively known as congenital ZIKV syndrome in newborns of infected pregnant women [1,2]. ZIKV infection impacts cells involved in fetal brain development; ZIKV RNA has been detected in human fetal brain tissue and placenta, and in vitro, ZIKV can infect placental and neuronal cells including neural progenitor cells [2,3,4,5].

The genomes of ZIKV and other flaviviruses encode three structural and seven nonstructural (NS) proteins. Upon infection, flaviviruses express viral proteins, replicate their RNA, and assemble virus particles in a ‘replication factory’ or ‘viroplasm’, an elaborate network of interconnected endoplasmic reticulum (ER) membranes [6,7,8,9,10]. Once the viral RNA is released from the virus particle after trafficking into the cell, ribosomes at the ER translate the RNA into a single polyprotein. The polyprotein is inserted into the ER membrane through its numerous transmembrane domains found in most ZIKV proteins except NS1, which localizes to the ER lumen, and NS3 and NS5, which are positioned on the cytoplasmic side of the ER membrane. After insertion into the ER membrane, the polyprotein is cleaved into individual proteins by host and ZIKV proteases. At the cytoplasmic side of the ER membrane, the NS proteins form a replication complex. This complex is responsible for viral RNA replication and processing which results in positive-sense single-stranded viral RNA. The RNA genome is then packaged into virus particles within the viroplasm.

Electron tomography and transmission electron microscopy imaging of flavivirus viroplasms have revealed the ultrastructures formed with ER membranes [6,7,8,9,10,11,12,13,14,15]. During replication of the flavivirus RNA, the ER membrane bends towards the lumen to form invaginations which retain a small pore facing the cytoplasm. The viral replication complex facilitates the formation of these structures, where the viral RNA is replicated and processed on the cytoplasmic side of the ER within these vesicular invaginations. The replicated virus RNA is thought to be released through the pore, and it is then incorporated into budding ER sites that contain the structural proteins for the assembly of virus particles. Connected to the sites of the ER that contain these invagination structures, the viroplasm also contains multiple collapsed ER membranes called convoluted membranes. Convoluted membranes are thought to be sites enriched in viral polyprotein processing [6,9,11].

These studies reveal that the viroplasm is an ER membrane-derived compartment where the critical steps of virus replication and assembly occur. How the compartment forms and becomes distinguished as a discrete compartment is not clear. In addition, it is not clear how the compartment boundary is formed. The ZIKV viroplasm has not been described to have a bounding membrane and can instead be another example of protein condensate-driven compartment assembly [16]. Resolution of mechanisms for viroplasm compartment formation are yet to be understood.

Microtubules (MTs) serve many essential roles in the cell including mitotic spindle assembly, intracellular trafficking, and cell morphology. MTs also support flavivirus infection; MT arrays localize at the viroplasms of different flaviviruses including those from ZIKV, DENV, Kunjin virus, and tick-borne encephalitis virus [15,17,18,19,20,21,22,23]. Through MT depolymerization or stabilization, drug manipulation of the MT array can affect ZIKV virus particle trafficking to the ER and virus production [15,22,23,24,25,26,27]. Moreover, the knockdown of the MT plus-end protein EB3 inhibits flavivirus entry into the cell [27].

MTs are organized by diverse microtubule-organizing centers (MTOCs) that vary by cell type [28,29,30,31,32,33,34,35,36,37,38,39,40,41]. Present in most animal cells, the centrosome is the best understood MTOC. It contains a pair of centrioles surrounded by a pericentriolar material (PCM) where MT assembly occurs through the MT nucleator gamma-tubulin and other MT regulators; the centrosome has mitotic and non-mitotic roles that affect human development [28,29,32,33,34,35,36]. Mutations in any one of at least 16 centrosomal protein-encoded genes result in a spectrum of disorders that cause developmental microcephaly (autosomal recessive primary microcephaly/MCPH) and primordial dwarfisms (Seckel syndrome/SCKL and others) [28,42,43,44,45,46,47,48,49]. However, the mechanisms linking centrosome dysfunction to these developmental disorders might differ depending on the gene involved [28,42,43,44,45,46,47,48,49]. In addition to the centrosome, non-centrosomal MTOCs (ncMTOCs) organize MTs at various sites in the cell to support a range of cellular functions [30,31,32,33,34,35,36,37,38,39,40,41]. The Golgi apparatus is the predominant ncMTOC in many mammalian cells and requires AKAP450, encoded by *AKAP9*, for its MTOC function in mammalian cells, and the Golgi MTOC regulates polarized cell migration and secretion [34,35,36,39,40,41,50,51,52,53,54,55,56,57]. In the absence of the centrosome and the Golgi MTOC in mammalian cells, cytoplasmic complexes comprised of PCM proteins including pericentrin and gamma-tubulin generate MTs [58]. 

In addition to requiring host MTs for the viral infection cycle, proteins encoded by a wide range of viruses impact MT assembly and also centrosome duplication and function [59,60,61,62,63,64,65,66,67]. ZIKV adversely affects centrosome organization in culture and in vivo by disrupting the centriole’s structure, reducing levels of some centrosomal proteins at the centrosome, and causing centrosomal amplification [68,69,70,71,72,73]. In addition, the centrosome has been shown to associate with the ZIKV viroplasm [15]. The effect of viruses on the Golgi MTOC and their reliance on it is still unknown. However, the Golgi MTOC in human cytomegalovirus infected cells was recently shown to regulate nuclear rotation to facilitate cell migration during infection and viral replication [67]. The requirement of MTOCs for ZIKV or other flaviviruses infection cycles has not yet been established.

Here we report that ZIKV forms a toroidal-shaped viroplasm to which MTs, the centrosome, and the Golgi MTOC closely associate. We show that MTs are required for viroplasm organization and the viral infection cycle through virus production. Although the viroplasm organizes around the cell’s major MTOCs, loss of both MTOCs does not affect virus production or viroplasm formation. However, the centrosome is primarily responsible for the viroplasm’s toroidal structure. In addition, MTs are still anchored to the surface of the viroplasms during ZIKV infection in cells without centrosomes and impaired Golgi MTOCs, and we propose that the viroplasm is a site of MT organization through MT anchoring. 

## 2. Materials and Methods

### 2.1. Cell Culture

We used SNB19 cells (Charles River Laboratories, Inc. under contract of the Biological Testing Branch of the National Cancer Institute), a derivative from the U-251 cell line, for viral and mock infections, and they were cultured in the RPMI medium (Cytiva-HyClone, cat# SH30027.02). Vero E6 cells (ATCC) were used for virus stock production and the focus forming assay, and they were cultured in the DMEM media (Corning, cat # 10-0170-CV). Both cell media were supplemented with 10% FBS (Avantor Seradigm, cat# 97068-85) and PenStrep (Corning, cat# 30-002-CI). All cells were tested for and free of mycoplasma using the PCR Mycoplasma Detection Kit (SouthernBiotech, cat# 13100-01). 

### 2.2. Drug Treatments

To eliminate centrosomes from SNB19 cells, cultures were treated for 7 days with media containing 125 nM centrinone [74] (Tocris, cat# 5687) diluted from a 125 μM stock in DMSO. At 7 days of centrinone treatment, centrosomes were eliminated in approximately 90% of the cells. To remove MTs in SNB19 cells, cultures were treated with media containing 10 μM nocodazole (noc) (Sigma-Aldrich, cat# M1404) diluted from a 33.2 mM stock in DMSO for the indicated times. To arrest cells in S-phase, cells underwent a double thymidine block before infection as described previously [75].

### 2.3. Virus Stock Production and Infections

We used the following virus strains: ZIKV-MR766 (ZeptoMetrix), ZIKV-PRVABC59 (ATCC), and DENV-2 (a kind gift from Qianjun Li, University of Alabama, Birmingham). Virus stocks were made as previously described [76]. In short, Vero E6 cells were infected using virus diluted into media at a multiplicity of infection (MOI) of 0.01 and incubated with cells for 2 h. Viral media was then replaced with fresh media, and the supernatant was collected 72–96 h post-infection (p.i.) when the cytopathic effect (CPE) was detected in a majority of the cells. Supernatants were then centrifuged at 1000× *g* for 10 min, filtered using a 0.45-micron filter, and stored at −80 °C. 

**Figure 1:**
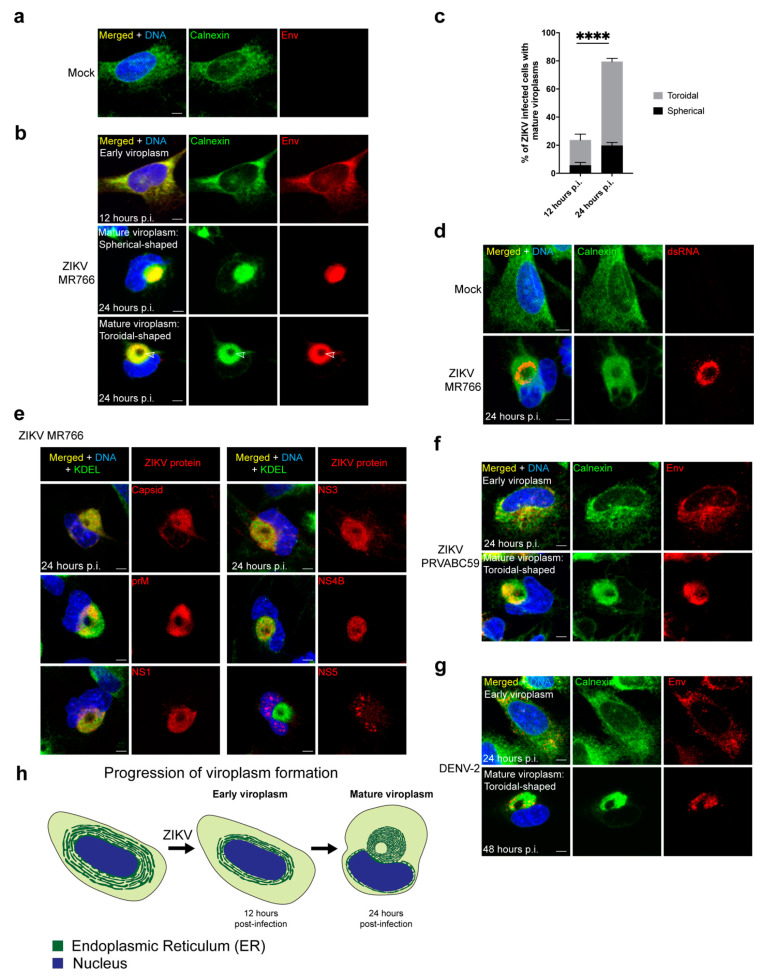
ZIKV forms a toroidal-shaped viroplasm: (**a**,**b**) Immunofluorescent (IF) staining of mock (**a**) and ZIKV-infected (**b**); Ugandan isolate (MR766) SNB19 cells fixed 12 h and 24 h post-infection (p.i.). ZIKV protein envelope (Env; red) localizes to the ER membrane marked with calnexin (green). (**b**) Upper panel shows example of an early viroplasm. Middle and bottom panels show examples of mature viroplasms that are spherical and toroidal-shaped, respectively. Arrowheads indicate the core of the viroplasm; (**c**) quantification of mature viroplasms for ZIKV infected cells at 12 and 24 h p.i. T-test comparing the frequency of mature viroplasms for the following conditions from five independent experiments (15–47 imaged cells/experiment), infected cells 12 h p.i. vs. 24 h p.i. (*p*-value < 0.0001, ****). Black and grey sections of the bar graph represent the percentage of ZIKV infected cells with spherical-shaped and toroidal-shaped viroplasms, respectively. Significance was assessed using an unpaired two-tailed Student’s *t*-test. Error bars are means ± s.e.m.; (**d**) IF staining of dsRNA (red) localization at the mature viroplasm as marked by calnexin (green) in mock- and ZIKV-infected cells. The mature viroplasm image is also shown in S1c including XZ and YZ cross-sections of the Env and dsRNA staining; (**e**) IF staining of ZIKV-infected cells at 24 h p.i. Mature viroplasms are marked with ER marker KDEL (green) and individual ZIKV proteins (red); (**f**,**g**) IF staining of Puerto ZIKV Rican isolate (PRVABC59), (**f**) infected cells fixed 24 h p.i. and dengue serotype 2 (DENV-2), (**g**) infected cells fixed 24 and 48 h p.i. The early and mature toroidal-shaped viroplasms are marked by calnexin (green) and Env (red); (**h**) illustration showing ER rearrangements during infection that lead to viroplasm development; for all IF staining, DAPI labels the nucleus (blue). Scale bars: 5 μm.

For infections with SNB19 cells, cultures were incubated in virus diluted in culture media at a MOI of 5 for the ZIKV infection time course experiment (Figure 1b,c and Figure S1b) and 1 for all other ZIKV infection experiments or media alone for mock infection for 2 h, and then viral media was exchanged with fresh media. Cells were then fixed using methanol fixation after the indicated times (12 h, 24 h, 48 h p.i.). During infections of SNB19 cells that we would measure viral infection and viral production, SNB19 cells were rinsed two times with serum-free media after infection to remove the residual virus. To quantify virus stocks and SNB19 cell supernatants after viral infection, we used the focus forming assay [77]. Briefly, we diluted viral supernatants or stocks 3–6 fold and applied them to Vero E6 cells in duplicate. For the cell supernatant collected from control and noc-treated infected SNB19 cells, we supplemented the control supernatant with equal concentrations of noc as the noc-treated supernatant and proceeded with dilutions as listed above.

After 2 h, media from viral dilutions was removed, and an overlay of DMEM-methylcellulose was applied to the cells. After 48 h p.i., we fixed the cells with 4% PFA PBS solution. We stained for the ZIKV envelope protein, and we used the DAB Substrate kit (Vector Laboratories, Cat# SK-4100) to visualize foci to count foci number and calculate viral titers. 

### 2.4. MT Regrowth Assays

SNB19 cells were treated with noc for 1–2 h. They were then rinsed three times with ice-cold media, and cells were given warm (37 °C) media and placed on a pre-warmed (37 °C) metal block for the indicated times of MT regrowth. Media was then removed, and cold methanol was added to fix cells for 10 min at −20 °C. Cells then were rinsed 4–5 times with PBS to remove residual methanol. 

### 2.5. Generation of AKAP450 KO SNB19 Lines

AKAP450 (gene: *AKAP9*) KO lines were generated using CRISPR-Cas9 mediated genome editing as described previously [51,78]. Briefly, we cloned the guide RNA [51] into the plasmid pSpCas9(BB)-2A-Puro (PX459) V2.0 (Addgene, plasmid #62988, [78]) and transfected the plasmid using Lipofectamine 3000 (Invitrogen, cat# L30000-15) into SNB19 cells. One day after transfection, the cells were treated for 3 days with media containing 2 μg/ml puromycin diluted from a 2 mg/ml stock in water and then replaced with regular media. After expanding the selected culture, we then isolated cells by diluting culture to 0.5 cells per well in media and distributed the cells into wells of four 96-well plates. We screened 12 clones for protein knockout using IF staining against AKAP450. We further characterized two lines: clonal lines 7 and 9 (Figure S4a–c). Western blotting and IF staining probed for AKAP450 showed complete knockout of protein levels for the two lines (Figure S4a,b). We also performed MT regrowth assays on the two clonal lines and tested rescue by transfection with a plasmid that expressed AKAP450-GFP (a kind gift from Anna Akhmanova [51]) (Figure S4c). This experiment confirmed the loss of the Golgi MTOC activity in both KO lines as MTs did not regrow from the Golgi after 3 min of noc recovery (Figure S4c). In addition, the MT organization ability is due to AKAP450 alone as ectopic AKAP450 expression in both KO lines rescued MTOC activity (Figure S4c). We used clonal line 9 for all subsequent experiments.

### 2.6. RNA Isolation, Reverse Transcription-Quantitative PCR (RT-qPCR), and Data Analysis

Total RNA of cell pellets and cell culture supernatants were isolated by TRIzol^TM^ (Invitrogen, cat# 15596026) or the TRIzol^TM^ LS reagent (Invitrogen, cat# 10296010), respectively, and quantified by nanodrop. Less than 1 μg of total RNA was used for RT by qScript cDNA SuperMix (Quantabio, cat# 95048). A qPCR was performed following the protocol as described previously [79,80]. The standard 3-step cycles protocol (40 cycles of 95 °C for 5 s, 60 °C for 10 s, 72 °C for 20 s) was used instead of the one-step fast 2-step cycles as the input was cDNA and not RNA. PerfeCTa SYBR Green FastMix (Quantabio, cat# 95072), assay primers (GAPDH-F, GGAGCGAGATCCCTCCAAAAT; GAPDH-R, GGCTGTTGTCATACTTCTCATGG; ZIKV-3′-F, AGGATCATAGGTGATGAAGAAAAGT; ZIKV-3′-R, CCTGACAACATTAAGATTGGTGC), and cDNA were prepared in 20 μL reaction and run on CFX96 qPCR machine. ZIKV RNA levels in the cell pellet experiments were first normalized to GAPDH and then were compared with the control group with the ΔΔCt method. ZIKV RNA levels in the cell supernatant experiments were directly normalized to control samples. 

### 2.7. Immunofluorescent (IF) Staining and Microscopy

For IF staining, the cells were seeded onto 18 mm circular coverslips in 12-well plates. To fix, the cells were rinsed once with PBS and then incubated in cold methanol for 10 min at −20 °C. The cells were then rinsed 4–5 times with PBS to remove residual methanol. We stained with primary antibodies for 1–2 h at room temperature or overnight at 4 °C, then with secondary antibodies and DAPI (Invitrogen, cat# D1306) for 1 h at room temperature. The antibodies were diluted in a PBS solution containing 5 mg/mL BSA (Sigma-Aldrich, cat# A2058) and 0.1% saponin (Sigma-Aldrich, cat# 47036). Cells were rinsed four times for 5 min with PBS after primary and secondary antibody incubations and mounted after secondary antibody wash. 

Samples were imaged on a Nikon A1 confocal microscope with a 60×NA 1.49 oil immersion objective. We used the NIS-elements (Nikon) software for imaging and image processing. For live cell imaging, we generated stable cell lines expressing EB3-mApple in WT and AKAP450 KO SNB19 cells. To create the lines, we transfected the cells with pQCXP-EB3-mApple [81], selected for transfected cells using 2 μg/mL puromycin, and expanded cultures. For live cell imaging, the cells were treated +/− centrinone, seeded onto glass-bottom dishes, +/− ZIKV infected, and imaged 24 h p.i. We used an ER tracker™ Blue-White DPX (Invitrogen, cat# E12353) to label the ER which allowed us to identify infected cells with mature viroplasms for imaging.

### 2.8. Antibodies

The following primary antibodies were used: mouse monoclonal anti-flavivirus group antigen (envelope) (D1-4G2-4-15, cat# MAB10216, Millipore, 1:1000 for IF), rabbit polyclonal ZIKV envelope protein (cat# EFS001, Kerafast, 1:1000 for IF), rabbit monoclonal anti-calnexin (C5C9, cat# 2679S, Cell Signaling, 1:100 for IF), mouse monoclonal anti-KDEL (10C3, cat# ADI-SPA-827, Enzo Life Sciences, 1:500 for IF), mouse monoclonal anti-double-stranded(ds) RNA (rJ2, cat# MABE1134, Millipore, 1:200 for IF), rabbit polyclonal anti-capsid (cat# GTX133317, GeneTex, 1:500 for IF), rabbit polyclonal anti-NS4B (cat# GTX133311, GeneTex, 1:1000 for IF), rabbit polyclonal anti-NS5 (cat# GTX133312, GeneTex, 1:500 for IF), rabbit polyclonal anti-NS3 (cat# GTX133309, GeneTex, 1:200 for IF), rabbit polyclonal anti-precursor membrane (prM) (cat# GTX133305, GeneTex, 1:500 for IF), rabbit polyclonal anti-NS1 (cat# GTX133307, GeneTex, 1:4000 for IF), mouse monoclonal anti-acetylated tubulin (6-11b-1, cat# T6793, Sigma-Aldrich, 1:2000 for IF), rat monoclonal anti-tyrosinated alpha-tubulin (YL1/2, cat# MA1-80017, Invitrogen, 1:1000 for IF), mouse monoclonal anti-alpha-tubulin (DM1a, cat# T9026, Sigma-Aldrich, 1:20,000 for western), mouse monoclonal anti-centrin (20H5, cat# 04-1624, Millipore, 1:1000 for IF), rabbit polyclonal anti-CEP192 (cat# A302-324A, Bethyl labs, 1:2000 for IF), rabbit polyclonal anti-CEP152 (cat# A302-480A, Bethyl labs, 1:2000 for IF), rabbit monoclonal anti-GM130 (D6B1, cat #12480, Cell Signaling, 1:3000 for IF), mouse monoclonal anti-AKAP450 (Clone 7, cat # 611518, BD Biosciences, 1:1000 for IF, 1:500 for western), mouse monoclonal anti-EB1 (Clone 5, cat# 610535, BD Biosciences, 1:1000 for IF), chicken anti-cenexin (a kind gift from Eugene Xu, 1:2000 for IF), and mouse monoclonal anti-GCP2 (Clone 01, a kind gift from Pavel Dráber [82], 1:500 for IF). We used Alexa Fluor^TM^ 488, 568, and 647 conjugated goat secondary antibodies (Invitrogen, 1:1000) for IF staining and IRDye800CW Goat anti-mouse secondary antibody (LI-COR, 1:20,000) for western blotting. We used peroxidase AffiniPure Goat anti-mouse secondary antibody (H+L) (cat# 115-035-003, Jackson ImmunoResearch, 1:10,000) for the focus forming assay.

### 2.9. Centrosome Intensity Quantification and Statistics

The protein’s intensity at the centrosome was determined by measuring the total fluorescence at the centrosome from a standard region of interest around the centrosome and subtracting out the background fluorescence [83]. The intensity was measured using the NIS-elements (Nikon) software. For all quantifications, GraphPad Prism was used to form graphs and perform unpaired two-tailed Student’s *t*-tests to determine statistical significance. 

## 3. Results

### 3.1. ZIKV Reorganizes the ER into a Compact Toroidal-Shaped Viroplasm

ZIKV undergoes virus particle assembly within a subcellular compartment referred to as the viroplasm, which is derived from the ER during infection by flaviviruses [6,7,8,9,10]. During infection of SNB19 cells, a human astrocytoma line, with the Ugandan strain of ZIKV, the ER rearranges and condenses from a dispersed organelle, as seen in mock-infected cells, into a compact viroplasm (Figure 1a,b,h; Movie S1). In addition, although ZIKV causes S-phase arrest [3,75], the few cells we observe in mitosis appear to have normal bipolar spindles and cytokinesis (Figure S1a).

Over the course of 24 h, the ER undergoes a substantial reorganization during ZIKV infection. We detected changes to the ER morphology during ZIKV infection at 12 h p.i., in which the ER is still distributed around the nucleus but is more compact in infected cells compared with mock-infected cells (Figure 1a–c,h and Figure S1b). By 24 h p.i., the ER significantly reorganizes in a majority of infected cells into a mature viroplasm in which most of the ER integrates into a single large compartment located beside the nucleus (Figure 1a–c,h and Figure S1b; Movie S1). At 24 h p.i., the mature viroplasm is the prominent structure during ZIKV infection (79% of infected cells) compared with 12 h p.i. (23% of infected cells) (Figure 1a–c,h and Figure S1b). The mature viroplasm predominantly has a toroidal shape (60% of infected cells at 24 h p.i.) with a less frequent spherical shape (19% of infected cells at 24 h p.i.), and the nucleus is typically distorted into a concave ‘bean-like’ shape adjacent to the viroplasm (Figure 1a–e,h; Movie S1). The double-stranded RNA (dsRNA) of the ZIKV genome, a replication intermediate, occupies an internal subcompartment of the viroplasm and localizes close to the core (Figure 1d and Figure S1c). In addition, ZIKV structural (envelope, capsid, precursor membrane (prM)) and nonstructural proteins (NS1, NS3, NS4B) localize to the ER during ZIKV infection (Figure 1a,b,e and Figure S1b,d). When the viroplasm is mature at 24 h p.i., NS5 localizes to the nucleus (Figure 1e and Figure S1d), which was observed previously for NS5 during flavivirus infections [84]. 

We investigated whether the formation of the compact toroidal viroplasm induced by the ZIKV infection was a shared feature of other strains of ZIKV and DENV, a closely related flavivirus. We found that the Puerto Rican ZIKV isolate and DENV serotype 2 form similar toroidal viroplasms (Figure 1f,g). However, DENV appears to have a slower progression of infection as mature viroplasms appear at 48 h p.i. compared with the more rapid ZIKV viroplasm development within 24 h. 

### 3.2. MTs Reorganize at the Viroplasm during ZIKV Infection

**Figure 2:**
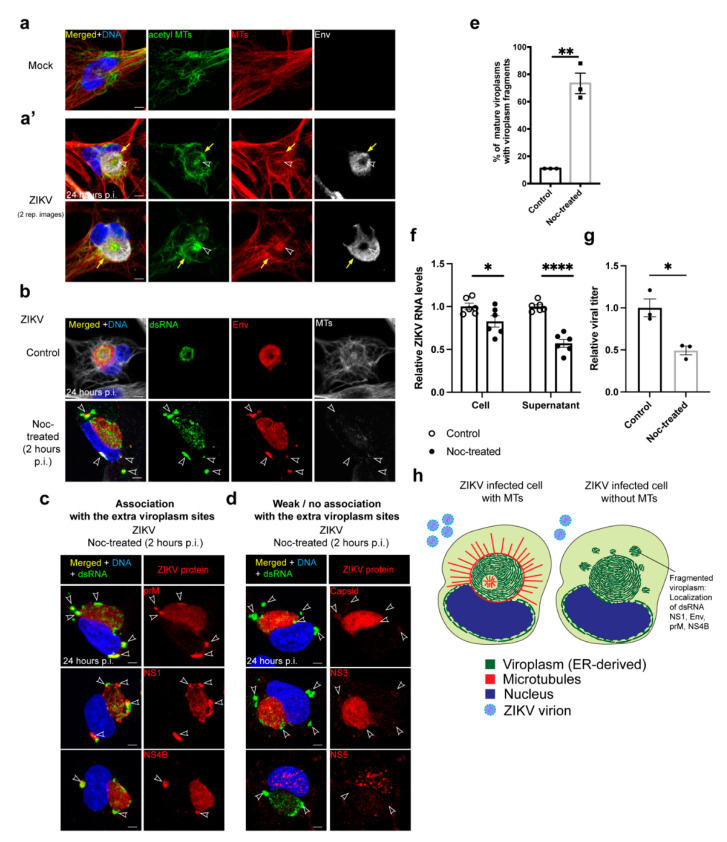
MTs are required for ZIKV viroplasm organization and efficient virus production (**a**,**a’**): IF staining of relatively stabilized acetylated (acetyl) MTs (green) and dynamic tyrosinated MTs (labeled MTs, red) of mock- and ZIKV-infected (MR766) SNB19 cells 24 h p.i. Viroplasms are marked with Env (white). Two representative images are shown for the MT cluster (indicated by arrowheads) at the core of the viroplasm. Arrows indicate MTs surrounding the viroplasm; (**b**) IF staining of control and nocodazole (noc)-treated cells 24 h p.i. dsRNA (green) and Env (red) marks ZIKV infected cells. Noc was added to cells 2 h p.i and remained in culture until fixation, and the MT array (white) is disrupted. White arrowheads point to extra viroplasm sites; (**c**,**d**) representative IF images to assess the localization of ZIKV proteins (red) to viroplasms contained for dsRNA (green) in noc-treated (2 h p.i.) ZIKV infected cells 24 h p.i. From two independent experiments (n = 18–31 imaged cells), (**c**) shows the ZIKV proteins that localize to the extra sites, and (**d**) shows the ZIKV proteins that are weakly or are not found at the satellite structures. They mostly localize to the mature main viroplasm (capsid, NS3) or the nucleus (NS5); (**e**) quantification of control and noc-treated ZIKV infected cells with mature viroplasms with viroplasm fragments as marked by dsRNA. *T*-test comparing the frequency of viroplasm fragments for the following conditions from three independent experiments (18–27 infected cells/experiment), infected: control vs. noc-treated (*p*-value < 0.01, **); (**f**) quantification of normalized ZIKV genome RNA levels in cell pellets and supernatants (medium) of S-phase arrested control and noc-treated (2 h p.i.) ZIKV-infected cells. RNA levels were measured by Reverse Transcription-quantitative PCR (RT-qPCR). T-tests comparing the viral RNA levels for the following conditions from six independent experiments, infected cell pellet: control vs. noc-treated (*p*-value = 0.043, *), infected supernatant: control vs. noc-treated (*p*-value < 0.0001, ****); (**g**) quantification of normalized focus forming units (FFU) per ml from collected supernatants of synchronized and S-phase arrested control and noc-treated (2 h p.i.) ZIKV infected cells. FFU per ml was measured with a focus forming assay. T-test comparing the viral titers for the following conditions from three independent experiments, infected: control vs. noc-treated (*p*-value = 0.013, *); for (**f**,**g**), individual experiments were normalized to mock-infected combined means; (**h**) illustration depicting ZIKV viroplasm organization in cells with and without MTs; significance was assessed using an unpaired two-tailed Student’s *t*-test. Error bars are means ± s.e.m.; for all IF staining, DAPI labels the nucleus (blue). Scale bars: 5 μm.

The expansive MT network in mock-infected cells rearranges to form a cage-like structure around the viroplasm that includes both dynamic tyrosinated and more stabilized acetylated [85] MTs (Figure 2a,a’). In addition, the core of the toroidal-shaped viroplasm contains a MT cluster that consists of both types of modified tubulins and varies in size (Figure 2a’). These MT reorganizations that occur in coordination with viroplasm assembly led us to investigate the roles of MTs on the ZIKV viroplasm formation and structure.

### 3.3. MTs Are Necessary for ZIKV Viroplasm Organization 

To determine how the global loss of MTs affects the development of the viroplasm, we used the MT-depolymerizing drug nocodazole (noc) to remove MTs. MTs are reported to have a role in ZIKV virion trafficking into the cell [22,23]. To circumvent this early requirement for MTs and assess the requirement of MTs for viroplasm assembly, we compared treatments of cells with noc for 1 h before infection, and 2 or 12 h p.i. Unexpectedly, we found that the viroplasm assembles after 24 h p.i. following all timings of noc treatment, indicating that ZIKV trafficking into the cell and viroplasm assembly are not strictly dependent on MTs in SNB19 cells (Figure S2a).

In cells treated with noc during ZIKV infection, a spherical viroplasm forms; however, its assembly is overtly perturbed including the loss of the toroidal shape (Figure 2b–e,h and Figure S2a,b). Smaller viroplasm-ER fragments also form when MTs are disrupted (Figure 2b–e,h and Figure S2a,b). Most of the ZIKV proteins that we evaluated (prM, NS1, NS4B, capsid, NS3, and envelope), and dsRNA, localize to the main viroplasm independent of MTs except for NS5, which localizes predominantly to the nucleus at 24 h p.i. regardless of noc treatment (Figure 1b,d,e, Figure 2b–d, and Figure S1d). In addition, the ZIKV envelope, NS1, prM, NS4B, and the dsRNA are bound at the smaller viroplasm fragments adjacent to the main viroplasm and throughout the cytoplasm (Figure 2b,c,e,h and Figure S2a,b). MT disruption greatly affects viroplasm organization as these extra viroplasm fragments (using dsRNA as a marker) are present in 73% of ZIKV infected cells with mature viroplasms without MTs compared with 11% in cells with MTs (Figure 2e). The dsRNA in cells without MTs is also dispersed throughout the main viroplasm compared with its localization to a viroplasm subcompartment adjacent to the core in cells with MTs (Figure 1d, Figure 2b–d, Figures S1c and S2a,b). Interestingly, ZIKV NS3 and capsid proteins were recruited or retained efficiently at viroplasms when MTs were disrupted, and they have little association with the viroplasm fragments in noc-treated cells (Figure 2d). These data show that MTs are necessary for viroplasm assembly or maintenance likely through the trafficking or retention of a subset of ZIKV proteins and the dsRNA at the mature viroplasm (Figure 2h). 

### 3.4. MTs Are Required for Efficient ZIKV Virus Production 

Because viroplasm organization and the localization of ZIKV proteins and dsRNA is disrupted by MT disassembly, we further examined the effect of noc on virus production. Since noc treatment arrests cells in mitosis, we arrested cells in the S-phase through a double thymidine block before infection and noc treatment. Arresting cells in the S-phase is not expected to impair the ZIKV infection cycle as virus production is enhanced in cells blocked in the S-phase [75]. 

Double thymidine treatment, however, had an impact on viroplasm organization. In addition to spherical viroplasms in double thymidine blocked control and noc-treated cells (Figure S2c), viroplasms are often poorly organized and surround the nucleus at 24 h after infection, an effect not common in asynchronous cell populations not subjected to double-thymidine treatment before ZIKV infection (Figure S2c’). These structures might be immature or aberrant viroplasms that form due to thymidine treatment or S-phase arrest.

To assay the effect of noc treatment on virus replication, we isolated RNA from cell pellets and supernatants at 24 h p.i., and measured ZIKV RNA levels intracellularly and in released virus particles, respectively, through a quantitative real-time PCR [79,80]. In noc-treated cells, there is a small 17% reduction in the amount of ZIKV RNA in cell pellets and a comparatively higher 43% reduction in ZIKV RNA in the supernatant (Figure 2f). In addition, cell supernatants from control and noc-treated cultures were collected, and the number of infectious virus particles produced was measured using the focus forming assay [77] (Figure 2g). Noc treatment results in a modest 50% reduction in virus particles produced in infected cells without MTs (Figure 2g,h). Together, these results indicate that MT-dependent ZIKV viroplasm organization is required for efficient virus production (Figure 2h).

### 3.5. The ZIKV Viroplasm Is Organized in Conjunction with the Centrosome and the Golgi MTOC 

**Figure 3:**
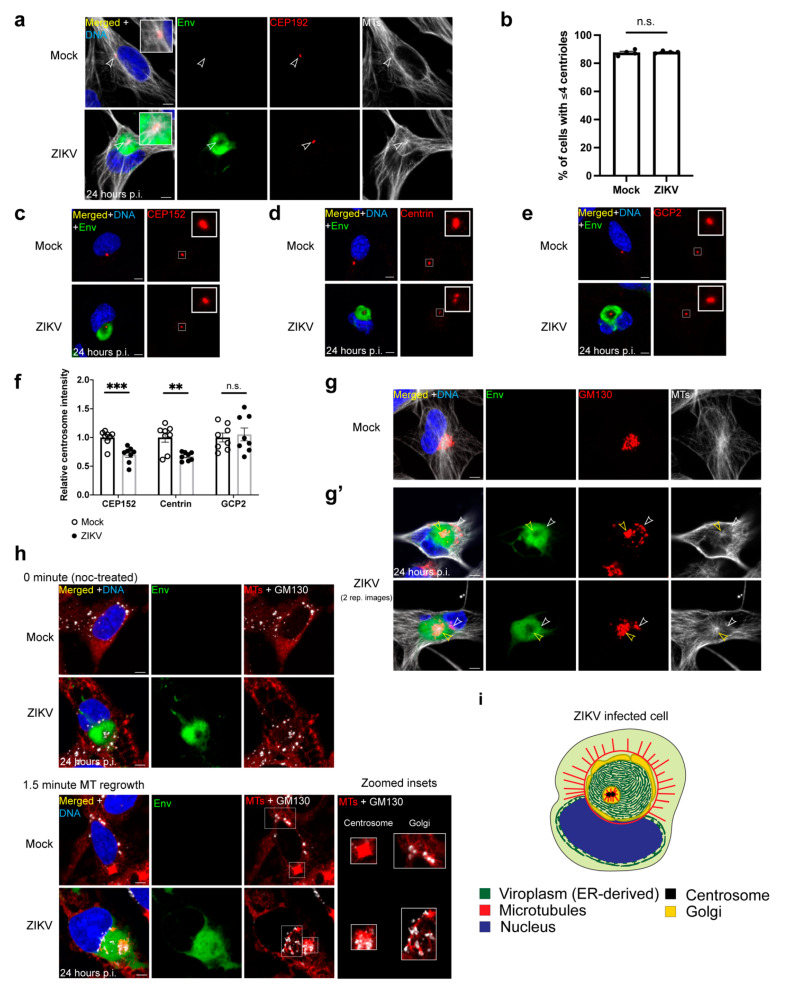
The centrosome and the Golgi MTOC associate with the ZIKV viroplasm, (**a**) IF staining for the centrosome protein CEP192 (red) in mock- and ZIKV (MR766)-infected SNB19 cells 24 h p.i. Viroplasms are marked by Env (green), and MTs are white. Insets show zoomed area where the centrosome is present, and arrowheads point to the centrosome; (**b**) quantification of the number of cells with 4 or fewer centrioles, measured by the number of centrin foci in mock- and ZIKV-infected cells 24 h p.i. T-test comparing centrosome number for the following conditions from four independent experiments (30–61 infected cells/experiment), mock vs. ZIKV (*p* = 0.67, n.s.); (**c**–**e**) representative images of the centrosomal proteins (red) CEP152 (**c**), centrin (**d**), and gamma-tubulin complex protein 2 (GCP2) (**e**) staining in mock- and ZIKV-infected cells 24 h p.i. Viroplasms are marked by Env (green); (**f**) normalized total intensity of centrosome proteins CEP152, centrin, and gamma-tubulin complex protein 2 (GCP2) in mock- and ZIKV-infected cells 24 h p.i. T-tests comparing mock- and ZIKV-infected intensity at the centrosome for the following centrosomal proteins from eight independent experiments (23–84 infected cells/experiment), CEP152: mock vs. ZIKV (*p*-value < 0.001, ***), centrin: mock vs. ZIKV (*p*-value < 0.01, **), GCP2: mock vs. ZIKV (*p*-value = 0.70, n.s.); (**g**,**g’**) IF staining of the Golgi protein GM130 (red) and MTs (white) in mock (**g**) and ZIKV-infected (**g’**) cells 24 h p.i. Viroplasms are marked by Env (green). Yellow arrowheads point to the Golgi at the core, and white arrowheads point to the Golgi surrounding the viroplasm; (**h**) MT regrowth assay. Noc-treated cells were imaged immediately (top) and after 1.5 min of regrowth (bottom) of mock- and ZIKV-infected cells. IF staining of Env (green), MTs (red), and GM130 (white). For the regrowth (bottom) panel, representative images of mock- and ZIKV-infected cells from two independent experiments that show MT regrowth (1–1.5 min) from the centrosome and the Golgi MTOC (mock, n = 15 imaged cells; ZIKV, n = 27 imaged cells). The right panel for regrowth shows zoomed insets of the centrosome and the Golgi MT regrowth; (**i**) illustration of ZIKV-infected cells depicting the localization of the centrosome, the Golgi, and the organization of MTs at the viroplasm; for (**b**,**f**), the mean percentage of cells with 4 or fewer centrioles (**b**) and mean centrosomal protein intensities (**f**) from each image were normalized to mock-infected combined means; significance was assessed using an unpaired two-tailed Student’s *t*-test. Error bars are means ± s.e.m.; for all IF staining, DAPI labels the nucleus (blue). Scale bars: 5 μm.

To investigate whether the requirements for MTs determined above rely on MTOCs, we next probed the involvement of the two major MTOCs in mammalian cells: the centrosome and the Golgi MTOC. We found that the centrosome is intimately associated with the viroplasm (95% of infected cells with mature viroplasms are associated with the centrosome) (Figure 3a,i and Figure S3a). When the viroplasm matures into the toroid structure, the centrosome localizes within the core of the toroid, an indication that it orchestrates the morphogenesis of this structure (Figure 3a,i and Figure S3a). 

There are normally one or two centrosomes (2 or 4 centrioles) in a cell, depending on the cell cycle stage. Centrosome amplification is a common feature of many viruses including ZIKV, and centrosome amplification is linked to mutations that cause microcephaly [59,60,61,62,63,64,69,70,71,86]. However, the numbers of centrioles in mock- and ZIKV-infected cells are not significantly different, indicating no effect by ZIKV on centrosome duplication in SNB19 cells (Figure 3b). Nevertheless, the levels of centriolar proteins centrin and CEP152 at the centrosome are significantly reduced by 33% and 30%, respectively, in ZIKV-infected cells (Figure 3c,d,f). However, we show no significant change in the levels of gamma-tubulin ring complex 2 (GCP2), a component of the MT nucleating gamma-tubulin ring complex, at the centrosome during ZIKV infection (Figure 3e,f). Therefore, ZIKV affects the centrosome but does not overtly impact its duplication or its MT assembly function.

**Figure 4:**
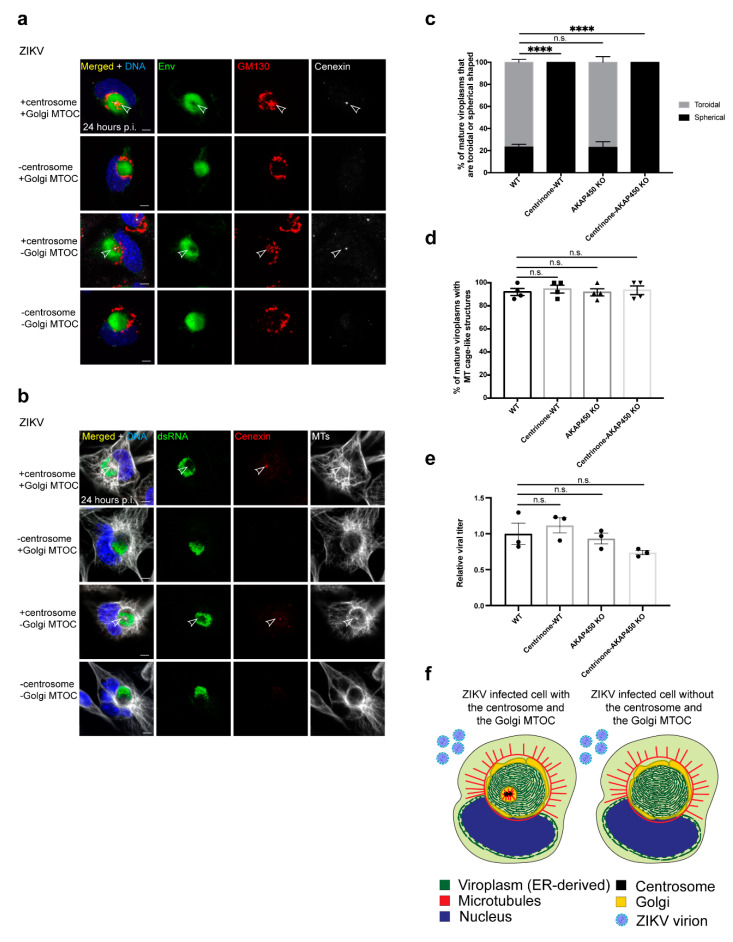
The centrosome and the Golgi MTOCs are not required for ZIKV proliferation (**a**,**b**) IF staining to show localization of Golgi protein GM130 ((**a**); red) and MTs ((**b**); white) of WT (+centrosome/+Golgi MTOC), centrinone-treated WT (-centrosome/+Golgi MTOC), AKAP450 KO (+centrosome/-Golgi MTOC), and centrinone-treated AKAP450 KO (-centrosome/-Golgi MTOC) ZIKV (MR766) infected SNB19 cells 24 h p.i. Viroplasms are marked by Env ((**a**); green) or dsRNA ((**b**); green). Centriolar protein cenexin ((**a**); white, (**b**); red) marks the centrosome, and fainter puncta are nonspecific signals. Arrowheads point to the centrosome and the Golgi (**a**) or MTs (**b**) at the core; (**c**) quantification of mature viroplasms that are toroidal or spherical-shaped in ZIKV-infected WT, centrinone-treated WT, AKAP450 KO, and centrinone-treated AKAP450 KO cells. T-tests comparing the frequency mature viroplasms that are spherical-shaped for the following conditions from four independent experiments (5–18 infected cells/experiment), infected: WT vs. centrinone-treated WT (*p*-value < 0.0001, ****), infected: WT vs. AKAP450 KO (*p*-value = 0.90, n.s.), infected: WT vs. centrinone-treated AKAP450 KO (*p*-value < 0.0001, ****); (**d**) quantification of mature viroplasms with MT cage-like structures in ZIKV-infected WT, centrinone-treated WT, AKAP450 KO, and centrinone-treated AKAP450 KO cells. T-tests of the frequency of the MT cage-like structure at mature viroplasms for the following conditions from four independent experiments (7–16 infected cells/experiment), infected: WT vs. centrinone-treated WT (*p*-value = 0.60, n.s.), infected: WT vs. AKAP450 KO (*p*-value = 0.96, n.s.), infected: WT vs. centrinone-treated AKAP450 KO (*p*-value = 0.77, n.s.); (**e**) quantification of normalized focus forming units (FFU) per ml from supernatants of ZIKV-infected WT, centrinone-treated WT, AKAP450 KO, and centrinone-treated AKAP450 KO cells. FFU per ml was measure with a focus forming assay. T-tests of the viral titer for the following conditions from three independent experiments, infected: WT vs. centrinone-treated WT (*p*-value = 0.56, n.s.), infected: WT vs. AKAP450 KO (*p*-value = 0.71, n.s.), infected: WT vs. centrinone-treated AKAP450 KO (*p*-value = 0.16, n.s.). Individual experiments were normalized to mock-infected combined means; (**f**) illustration of a ZIKV-infected cell without the centrosome and the Golgi MTOC compared with one with both MTOCs showing a MT cage and the loss of the toroidal viroplasm structure; Significance was assessed using an unpaired two-tailed Student’s *t*-test. Error bars are means ± s.e.m.; For all IF staining, DAPI labels the nucleus (blue). Scale bars: 5 μm.

During ZIKV infection, the Golgi reorganizes from a single contiguous unit as seen in mock-infected cells (Figure 3g) to a more dispersed structure associated with the mature viroplasm including the surface of the viroplasm and/or at the core together with the centrosome (100% of infected cells with mature viroplasms are associated with the Golgi) (Figure 3g’,i, Figure 4a and Figure S3a). Overall, the viroplasm organizes around the centrosome and the Golgi at the core and with the Golgi associated with the viroplasm surface during ZIKV infection (Figure 3i).

### 3.6. The Centrosome and the Golgi MTOC Nucleate the MTs Associated with the Viroplasm

We next performed an MT regrowth assay to determine the source of MTs in infected cells. Following MT disassembly by noc, MTs in cells grow back within minutes from the MTOCs after drug removal. In infected cells with both MTOCs intact, MT growth from the centrosome and the Golgi is readily detected after 1.5 min of regrowth (Figure 3h). We next performed live imaging of mock- and ZIKV-infected cells that express the MT plus-end tracking protein EB3 tagged with fluorescent mApple to monitor MT dynamics (Figure S3b,c’; Movies S2–S4). As expected, MTs grow from the centrosome and throughout the cytoplasm in mock-infected cells, where some growing MTs are attributed to the Golgi (marker not shown) (Figure S3b; Movie S2). In ZIKV-infected cells, MTs grow from the centrosome at the core of the viroplasm (Figure S3c; Movie S3) and from the MT cage-like structure on the viroplasm surface where the Golgi (marker not shown) often localizes (Figure 3g’ and Figure S3c,c’; Movies S3 and S4). Together, these data show that the centrosome and the Golgi are active MTOCs that organize MTs at the viroplasm in ZIKV-infected cells (Figure 3i).

### 3.7. The Centrosome Is Required to Form a Toroidal Viroplasm

As these two MTOCs associate with and generate MTs at the viroplasm, we tested the requirements of the centrosome and the Golgi MTOC for the organization of the ZIKV viroplasm (Figure 3i and Figure 4). To eliminate the centrosome from cells, cultures were treated with the PLK4-specific inhibitor centrinone, which efficiently blocks centriole replication, and cells lose centrioles by attrition at each division [74]. The Golgi MTOC requires AKAP450 for MTOC activity through its binding to the cis-Golgi protein GM130, and knockout of the *AKAP9* gene that encodes AKAP450 which blocks MTOC function at the Golgi [51,52]. We knocked out the *AKAP9* gene in SNB19 cells using CRISPR-Cas9 genome editing as has been performed previously in RPE1 cells [51,52], and we verified the knockout by IF staining and western blotting which probed for AKAP450 (Figure S4a,b). We further showed that mutant cells are deficient in Golgi MTOC activity and can be rescued by the expression of AKAP450-GFP (Figure S4c).

Following the removal of the centrosome and impairment of the Golgi MTOC function, together and separately, the ZIKV viroplasm still assembles into a spherical structure with no unincorporated viroplasm satellite structures as occurred with noc-treated cells (Figure 2b–e,h, and Figure 4a–c,f). Thus, the effects of noc treatment were more severe than the loss of both major MTOCs. However, there were structural differences in cells without centrosomes. The toroidal shape of the viroplasm and the localization of the Golgi and MTs to the center of the viroplasm is eliminated in ZIKV-infected cells without centrosomes (100% of infected cells with mature viroplasms without centrosomes are spherical, Figure 4a–c,f). Disruption of the Golgi MTOC, on the other hand, does not affect ZIKV viroplasm organization, as the toroidal shape is maintained in AKAP450 KO cells (Figure 4a). 

When both MTOCs were disrupted, there were no additional defects to the viroplasm beyond the loss of the core that is attributed to the removal of the centrosome (Figure 4a–c,f). In addition, the Golgi localization at the viroplasm surface does not require either MTOC as the Golgi still localizes there when the two MTOCs are eliminated (Figure 4a,f). Altogether, the centrosome is required for morphogenesis of the core of the viroplasm to generate its toroid structure and Golgi/MT recruitment to the core. However, neither MTOC is required to form a spherical viroplasm and the proper recruitment or maintenance of ZIKV proteins and dsRNA at the viroplasm (Figure 4a–c,f).

### 3.8. Virus Production IS Not Significantly Dependent on the Centrosome and Golgi MTOC

Whereas viroplasm assembly remains intact, albeit defective in cells without centrosomes, we next assessed whether production of infectious virus is affected when either or both MTOCs are impaired. To test this, we measured the amount of virus produced from infected cells at 24 h p.i. in control cells and cells without the centrosome and/or Golgi MTOC using the focus forming assay [77]. In contrast to the loss of MTs with noc treatment, there is no significant inhibition of infectious ZIKV virus production when the centrosome and the Golgi MTOC are impaired (Figure 4e). Interestingly, the centrosome and the Golgi together are not required to form the MT cage-like structure around the viroplasm as it is still present in infected cells with both MTOCs disrupted (Figure 4b,d,f). The MT cage-like structure is present in most WT cells with a mature viroplasm (92% of infected cells (WT)), and this frequency is not significantly different in cells without either or both MTOCs (Figure 4d). We propose that the viroplasm-associated MTs in infected cells without both MTOCs are sufficient to support viroplasm assembly and virus production (Figure 4f). 

### 3.9. MTs Are Anchored at the ZIKV Viroplasm 

**Figure 5:**
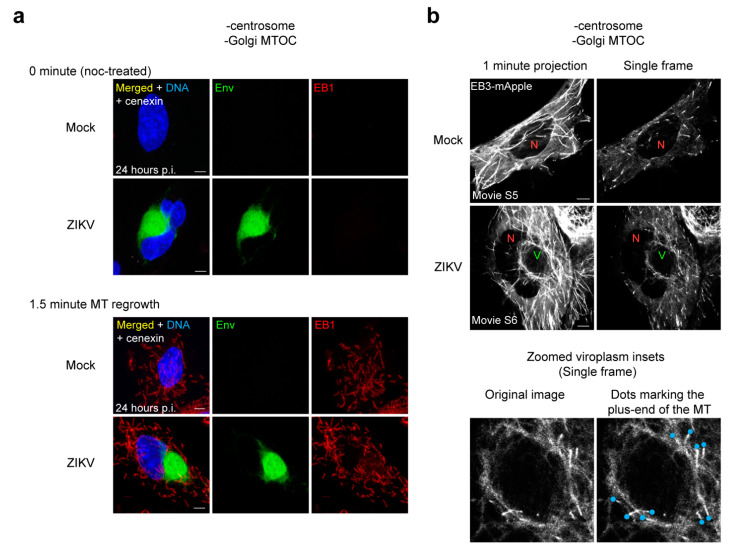
MTs are anchored at the ZIKV viroplasm (**a**) MT regrowth assay. A total of 0 min (noc-treated) and 1.5 min regrowth of centrinone-treated AKAP450 KO mock- and ZIKV(MR766)-infected SNB19 cells (-centrosome/-Golgi MTOC). Representative IF images of centrinone-treated AKAP450 KO mock- and ZIKV-infected cells from two independent experiments that show MTs as marked by MT-plus end protein EB1 regrowing randomly throughout the cytoplasm (mock, n = 8 imaged cells; ZIKV, n = 30 imaged cells). Viroplasms are marked by Env (green). DAPI labels the nucleus (blue); (**b**) Live imaging of MT-plus end protein EB3-mApple (white) of mock (Movie S5) and ZIKV-infected centrinone-treated AKAP450 KO (Movie S6) cells. The left panel shows 1 min projection of EB3 comets, and the right panel shows a single frame of the movie. Red ‘N’ indicates the nucleus, and green ‘V’ indicates the viroplasm. Zoomed inset of the viroplasm from a single frame of the movie shows EB3 comets (original image (left), marked image with blue dots off the growing plus-ends of MTs (right)) coming from the viroplasm. The cells were imaged for 1–2 min with 4 s per frame. Mock- and ZIKV-infected movies are representative of 10 and 24 movies, respectively, from four independent experiments that show similar MT dynamics in each condition; scale bars: 5 μm.

We found that in cells without the centrosome and the Golgi MTOC, a MT cage-like structure forms around the viroplasm suggesting the organization of MTs by an alternative process at the viroplasm (Figure 4b,d,f). To test this, we performed MT regrowth assays to determine the source of MTs in infected cells without the centrosome and the Golgi MTOC. In ZIKV-infected cells with both MTOCs, most MTs regrow from the Golgi and the centrosome (Figure 3h). In contrast, when both MTOCs are removed, MTs grow apparently randomly throughout the cytoplasm in ZIKV-infected cells, and MT regrowth is comparable with mock-infected cells (Figure 5a). Therefore, it appears that the viroplasm is not a site of MT nucleation despite a concentration of MTs there.

We next performed live imaging of MT plus-ends in cells using EB3-mApple to observe MT dynamics and track the origins of growing MTs in infected cells without both MTOCs (Figure 5b; Movies S5 and S6). Live imaging revealed that in cells with both MTOCs, dynamic MTs grow from the centrosome and around the ZIKV viroplasm (Figure S3b–c’; Movies S2–S4). In the absence of both MTOCs, some dynamic MTs grow from the MT cage-like structure in ZIKV-infected cells, whereas MTs appear to grow randomly throughout the cytoplasm in mock-infected cells (Figure 5b; Movies S5 and S6). These data indicate that MTs are anchored at the viroplasm during ZIKV infection. Therefore, we conclude that the surface of the ZIKV viroplasm anchors MTs, forming the MT cage-like structure where dynamic MTs are concentrated.

## 4. Discussion

Here we show that ZIKV infection is orchestrated through the assembly of a toroidal-shaped viroplasm in close association with the centrosome and Golgi MTOCs and where the centrosome is essential to form its core. MTs are organized in close proximity to the viroplasm, forming an MT cage-like structure surrounding it and a cluster of MTs at the core where the centrosome and some of the Golgi reside. Viroplasm organization requires MTs for its efficient morphogenesis and/or maintenance of viral dsRNA and a subset of ZIKV-encoded proteins. When MTs are disrupted, the amount of released virus particles is significantly reduced. Moreover, we further show that neither the centrosome nor the Golgi MTOCs is necessary for overall viroplasm formation or the viral infection cycle. However, the centrosome is essential for the normal morphogenesis of the viroplasm, contributing significantly to the formation of the core of the toroid structure. Still, the Golgi MTOC does not exhibit an overt requirement for its assembly. In the absence of MTOC activity from the centrosome and the Golgi, MTs are still organized at the viroplasm periphery, but those MTs are not nucleated there. Instead, we propose that they become anchored at the viroplasm periphery by an unknown virus and/or host factors. We propose that the surface of the ZIKV viroplasm contains a novel MTOC that operates by anchoring MTs there.

### 4.1. MT Requirements for ZIKV Viroplasm Organization and Virus Production

When MTs are disrupted during most of the infection cycle, the functional virus is produced, but at a reduced efficiency of about 50% which is consistent with other studies showing an MT requirement for ZIKV replication [24,25]. Nonetheless, it is remarkable that ZIKV proliferation has a high level of independence on MTs. On the other hand, studies looking at paclitaxel treatment, which stabilizes MTs, showed a much higher impediment on ZIKV replication [15,26]. Concerning how MTs affect the ZIKV infection cycle, we show that MT disruption has a small but significant effect on the completion of the entire cycle. We found a small reduction in the amount of cellular ZIKV RNA and a more considerable reduction in released ZIKV RNA and infectious virus particles in cells without MTs compared with control cells. This difference in cellular vs. released ZIKV RNA likely reflects the relative requirements of MTs for ZIKV RNA replication, which are small, and a higher reliance on MTs for virus assembly and/or trafficking of the virus out of the cell. Likely both of these effects are reflected in the reliance on MTs for proper viroplasm assembly. Nevertheless, cells can still produce infectious virus particles when MTs are disrupted, but perform it less efficiently.

While defective, we show that a ZIKV viroplasm still forms in cells without MTs. However, when MTs were disrupted, additional satellite sites of aggregated ER accumulate a subset of viral proteins and dsRNA. These extra sites of viroplasm material are either not maintained at the main viroplasm or fail to traffic correctly during viroplasm formation. These small satellite viroplasms and the loss of the MT cage-like structure around the viroplasm likely account for the loss of virus particle assembly and release in cells without MTs. Overall, we found that MTs play a role in viroplasm organization and viral production during ZIKV infection.

### 4.2. Impacts of ZIKV on the Centrosome

We found impacts to centrosome integrity reflected in the reduction in centriolar proteins at the centrosome. We and others have shown a significant reduction in MCPH/SCKL-linked protein CEP152 levels at the centrosome [68]. We also observed decreased centrin levels at the centrosome but no significant change in GCP2, which has an essential role in MT nucleation. ZIKV has also been shown to affect the centriole’s structure and the overall PCM organization [68,72,73]. The recessive inherited forms of microcephaly have links to centrosome dysfunction [28,42,43,44,45,46,47,48,49], and impacts to the centrosome can be a shared feature of MCPH/SCKL and ZIKV-linked microcephaly. Nevertheless, we show that the centrosome remains functional as an MTOC, and the centrosome serves as a central hub around which the viroplasm assembles. In addition, centrosome amplification is linked to the development of microcephaly [86]. While some studies have reported supernumerary centrosomes as a consequence of ZIKV infection [69,70,71], we did not detect changes in centrosome numbers in SNB19 cells consistent with reports by others [68,72,73], and so the impact of ZIKV on centriole amplification may be cell type dependent. In summary, we did not find an impact on centrosome replication or its MT assembly activity during ZIKV infection.

### 4.3. The Requirements of the Centrosome and Golgi MTOCs for ZIKV Infection

Both the centrosome and Golgi associate with the ZIKV viroplasm, and the centrosome significantly influences its morphology by generating the central core of the toroid structure. Therefore, it is surprising that neither of these two major MTOCs are essential for ZIKV viroplasm formation or the virus infection cycle. While the Golgi localizes to the viroplasm, the Golgi MTOC is not significantly involved in virus production. However, the Golgi MTOC might impact cellular activities of infected cells such as polarized cell migration and secretion, functions that are linked to the Golgi MTOC [34,35,36,39,40,41,50,51,52,53,54,55,56,57]. Furthermore, while the loss of the toroid core does not apparently affect virus production, the Golgi together with the centrosome are closely associated with the core, and this configuration may support virus proliferation. Recently, the tetraspanin CD63 was shown to regulate the production of ZIKV virus particles and also the secretion of infectious extracellular vesicles; when CD63 is depleted, the virus particle release is favored, whereas CD63 overexpression favors the secretion of infectious extracellular vesicles [80]. CD63 localizes to the viroplasm core [80], and potentially, the core has a role in modulating the relative contributions of the virus particles and infectious extracellular vesicles produced. Future work on the contributions of MTs and MTOCs to these two pathways for ZIKV infectious transmission may elucidate these functions. 

We and others have reported an MT cage-like structure at the ZIKV viroplasm [15]. Through this study, we were able to further determine the requirements for its formation as this MT structure still forms in the absence of both MTOCs. This MT structure likely plays a significant role in coordinating viroplasm organization and virus production independent of the centrosome and the Golgi MTOC. We propose that a mechanism exists for a ZIKV-induced organization of MTs at the viroplasm independent of the cell’s MTOCs. The MT regrowth assay showed that in mock-infected cells without both MTOCs, MTs grow back from random sites within the cytoplasm, as also shown by others [58], and this pattern was not altered in ZIKV-infected cells. However, MTs appear to be anchored at the viroplasm surface from static imaging of cells disrupted for both MTOCs, and live imaging with EB3-mApple indicates that the viroplasm is a major site of dynamic MTs. Potentially, ZIKV proteins and host MT proteins cooperate in organizing the MTs at the viroplasm independently of the centrosome and the Golgi MTOC. MT-associated and centrosomal proteins have been shown to interact with individual flavivirus proteins in interactome studies [87,88,89,90,91]. Flavivirus NS3 has also been shown to associate with MTs though it is unclear if this interaction is direct [92,93]. We propose that MTs are anchored at the viroplasm to form an MT cage-like structure, and a potential MTOC is formed through MT anchoring. However, the molecular mechanism for its organization and the involvement of virus and host proteins remains to be discovered.

### 4.4. Methods to Remove MTOCs to Test Viral Infection

MTs contribute to the infection process for a wide variety of virus classes including flaviviruses [22,23,65]. However, a comprehensive evaluation of the requirements of MTOCs on viral infection has not been investigated previously, and we show how the removal of the centrosome through the use of the drug centrinone [74] and disruption of Golgi MTOC function through the knockout of AKAP450 [51,52] affects ZIKV viral infection. These targeted methods for removing both MTOCs can be applied to test their requirements in the viral infection cycles of other classes of viruses.

## 5. Conclusions

In this study we found that ZIKV forms a toroidal-shaped viroplasm where MTs localize to the core and to the viroplasm surface. MTs play a role in ZIKV viroplasm organization and efficient virus production. In addition, the cell’s two major MTOCs, the centrosome and the Golgi, associate with the viroplasm. However, viroplasm assembly is not disrupted and virus production is not compromised when both MTOCs are removed. MTs and the centrosome are required to create the core of the viroplasm, whose functional contribution to ZIKV transmission remains speculative at present. In addition, MTs are anchored at the viroplasm surface in infected cells independently of the centrosome and Golgi MTOC, establishing a potentially novel ZIKV-dependent MTOC in this compartment. 

## Data Availability

The data presented in this study are available on request from the corresponding author.

## Supplementary Materials

The following are available online at https://www.mdpi.com/article/10.3390/cells10123335/s1, Figure S1: Viroplasm organization during ZIKV infection, Figure S2: Viroplasm organization following noc treatment at various times during ZIKV infection and also upon S-phase arrest, Figure S3: Association of the cell’s MTOCs with the ZIKV mature viroplasm and live imaging of EB3-mApple in mock- and ZIKV-infected WT SNB19 cells, Figure S4: Evaluation of AKAP450 (gene: *AKAP9*) KO SNB19 cells, Movie S1: 3D view of the ZIKV mature toroidal viroplasm, Movie S2: Live imaging of EB3-mApple in WT mock-infected SNB19 cells, Movie S3: Live imaging of EB3-mApple in WT ZIKV (MR766)-infected SNB19 cell imaged 24 h p.i. (Representative #1), Movie S4: Live imaging of EB3-mApple in WT ZIKV (MR766)-infected SNB19 cell imaged 24 h p.i. (Representative #2), Movie S5: Live imaging of EB3-mApple in centrinone-treated AKAP450 KO mock-infected SNB19 cells imaged 24 h p.i., Movie S6: Live imaging of EB3-mApple in centrinone-treated AKAP450 KO ZIKV (MR766)-infected SNB19 cells imaged 24 h p.i.
