# Supplementary material for: Coordination of Zika Virus Infection and Viroplasm Organization by Microtubules and Microtubule-Organizing Centers
Source: Cells. 2021 Nov 27;10(12):3335. doi: 10.3390/cells10123335 (PMC8699624; doi:10.3390/cells10123335)
Supplement: Supplementary file 1 [file cells-10-03335-s001.zip › cells-1398514-supplementary/Supplemental Materials.pdf]

Supplementary materials

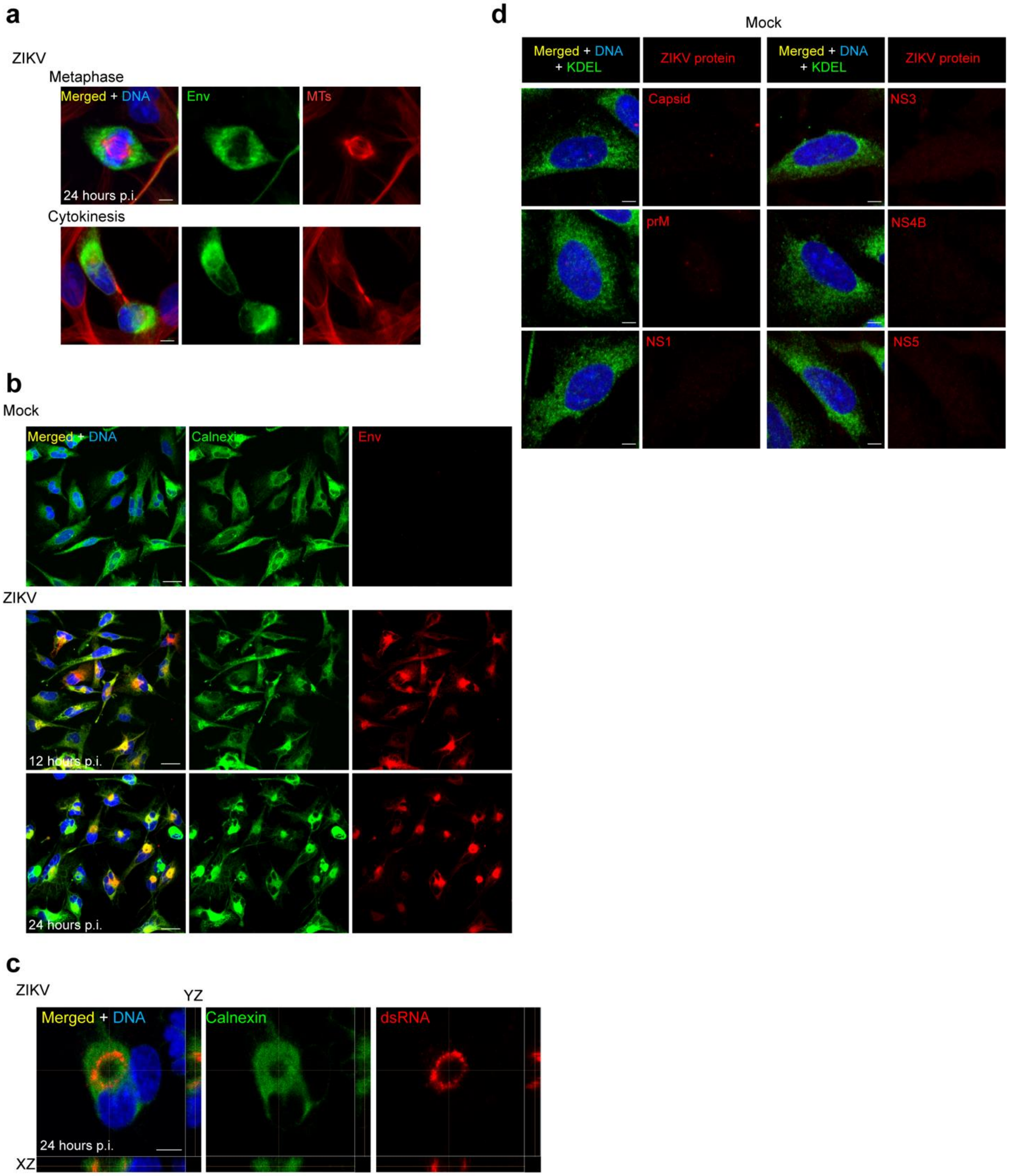

Figure S1: Viroplasm organization during ZIKV infection

a: IF staining of dividing ZIKV-infected (MR766) SNB19 cells examined 24 hours p.i. Env (green) marks infected cells, and MTs (red) show cells undergoing division.

b: IF staining of mock- and ZIKV(MR766)-infected cells at 12 hours and 24 hours p.i. Calnexin (green) and Env (red) show rearrangements to the ER during the progression of ZIKV infection.
c: Maximum projection image from Figure 1d which includes XZ and YZ cross-sections of IF staining of dsRNA (red) and calnexin (ER, green) in a ZIKV-infected SNB19 cell examined 24 hours p.i.
d: IF staining of ZIKV proteins (red) and KDEL (ER, green) in mock-infected cells.
For all IF staining, DAPI labels the nucleus (blue). Scale bars: 5 μm (**a,c,d**) and 25 μm (**b**).

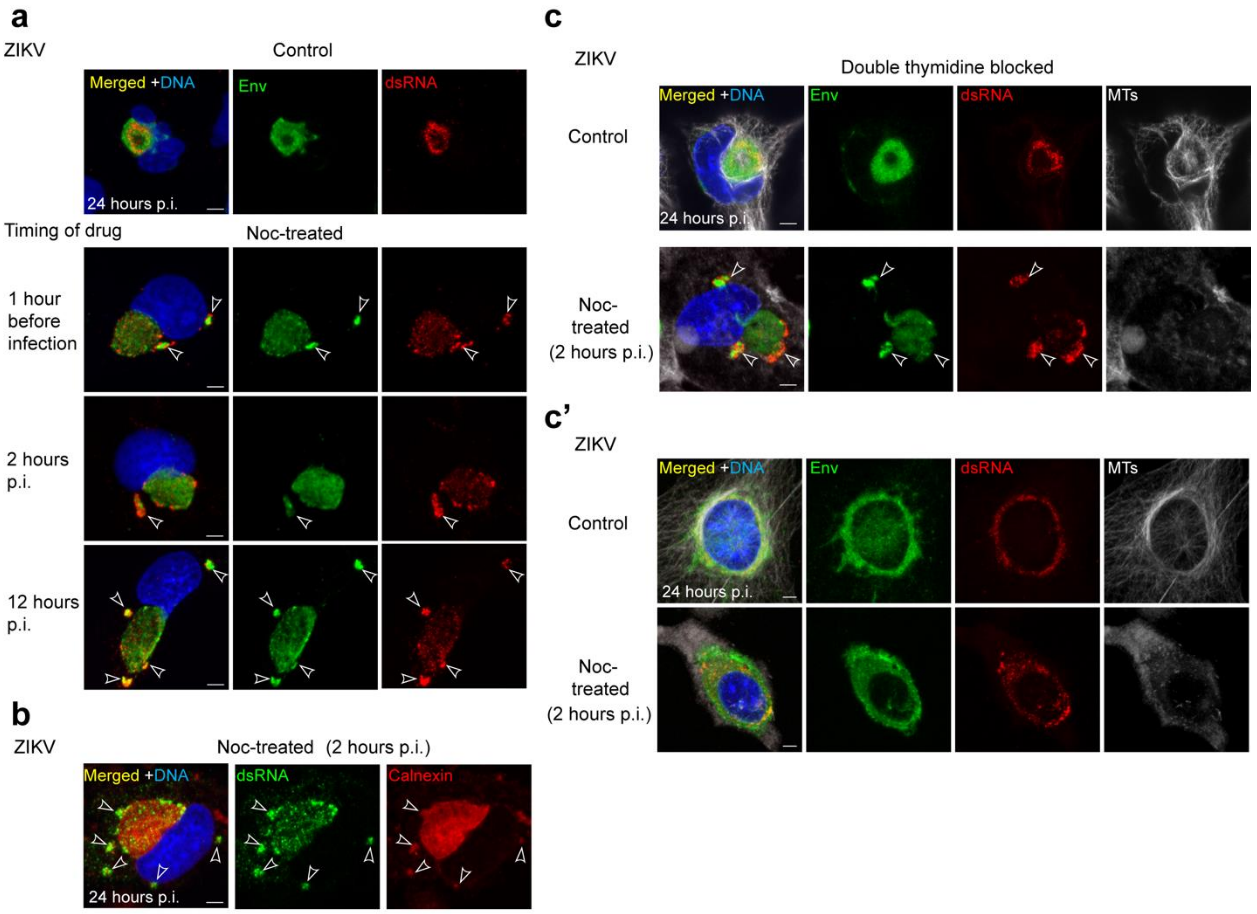

Figure S2: Viroplasm organization following noc treatment at various times during ZIKV infection and also upon S-phase arrest
(**a**)**:** IF staining of control and time-course noc-treated ZIKV-infected (MR766) SNB19 cells. The following timings of noc applications were performed: pretreated (1 hour before infection), 2 hours p.i. and 12 hours p.i. Viroplasms are marked with Env (green) and dsRNA (red). Cells were maintained in noc-containing media until fixed after 24 hours p.i.
(**b**)**:** IF staining of dsRNA (green) & ER marker calnexin (red) of ZIKV-infected noc-treated cells examined 24 hours p.i.
(**c,c'**)**:** IF staining of control and noc-treated ZIKV viroplasms from double thymidine blocked cells. Staining of infected cells marked by Env (green), dsRNA (red), and MTs (white).
For all IF staining, DAPI labels the nucleus (blue). Scale bars: 5 μm.

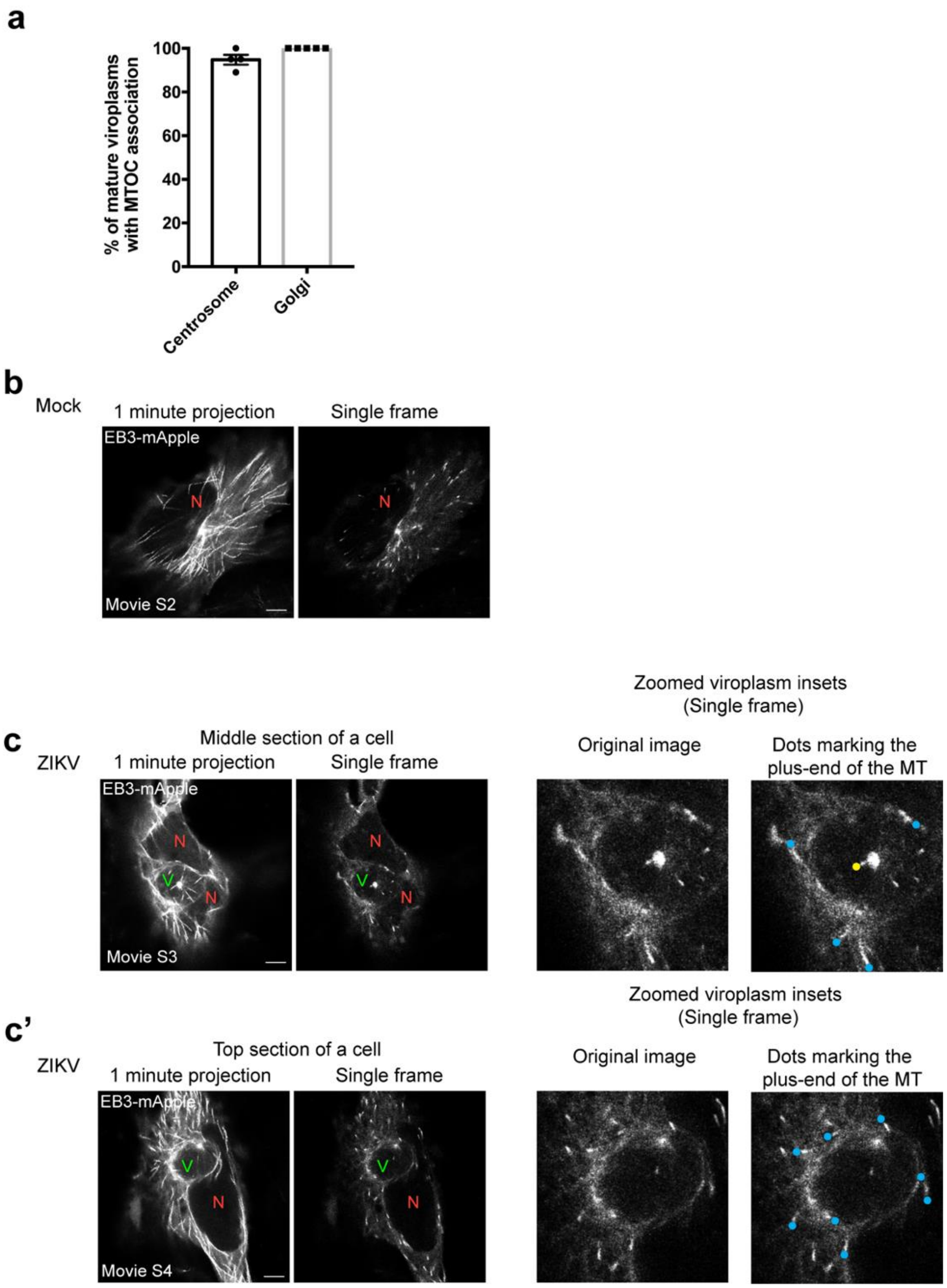

Figure S3: Association of the cell's MTOCs with the ZIKV mature viroplasm and live imaging of EB3-mApple in WT mock- and ZIKV-infected SNB19 cells

(**a**): Quantification of ZIKV infected cells that have MTOC association (centrosome or Golgi) with mature viroplasms at 24 hours p.i. Four (14-22 imaged cells/experiment), and five independent experiments (5-10 imaged cells/experiment) were quantified for the centrosome- and Golgi-associated mature viroplasms, respectively. Error bars are means ± s.e.m.

(**b-c'**)**:** The left panel shows a 1 minute projection of EB3 comets for WT mock- (**b**; from Movie S2) and ZIKV-infected (MR766) SNB19 cells (**c,c'**; from Movies S3, S4). The right panel shows a single frame of the movie for WT mock- (**b**) and ZIKV-infected cells (**c,c'**). Red 'N' indicates the nucleus, and green 'V' indicates the viroplasm. Two representative images are shown for ZIKV infected (**c,c'**) to show comets

from the centrosome (**c**) and the MT surrounding the viroplasm (**c,c'**). Zoomed insets of viroplasms from single frames of the movies show EB3 comets coming from around the viroplasm (original images (left) and marked image with blue dots off of the growing plus-ends of MTs (right)) and the centrosome (yellow dot off of the growing plus-end of a MT (right)). Cells were imaged for 2 minutes with 4 seconds per frame. Scale bars: 5 μm.

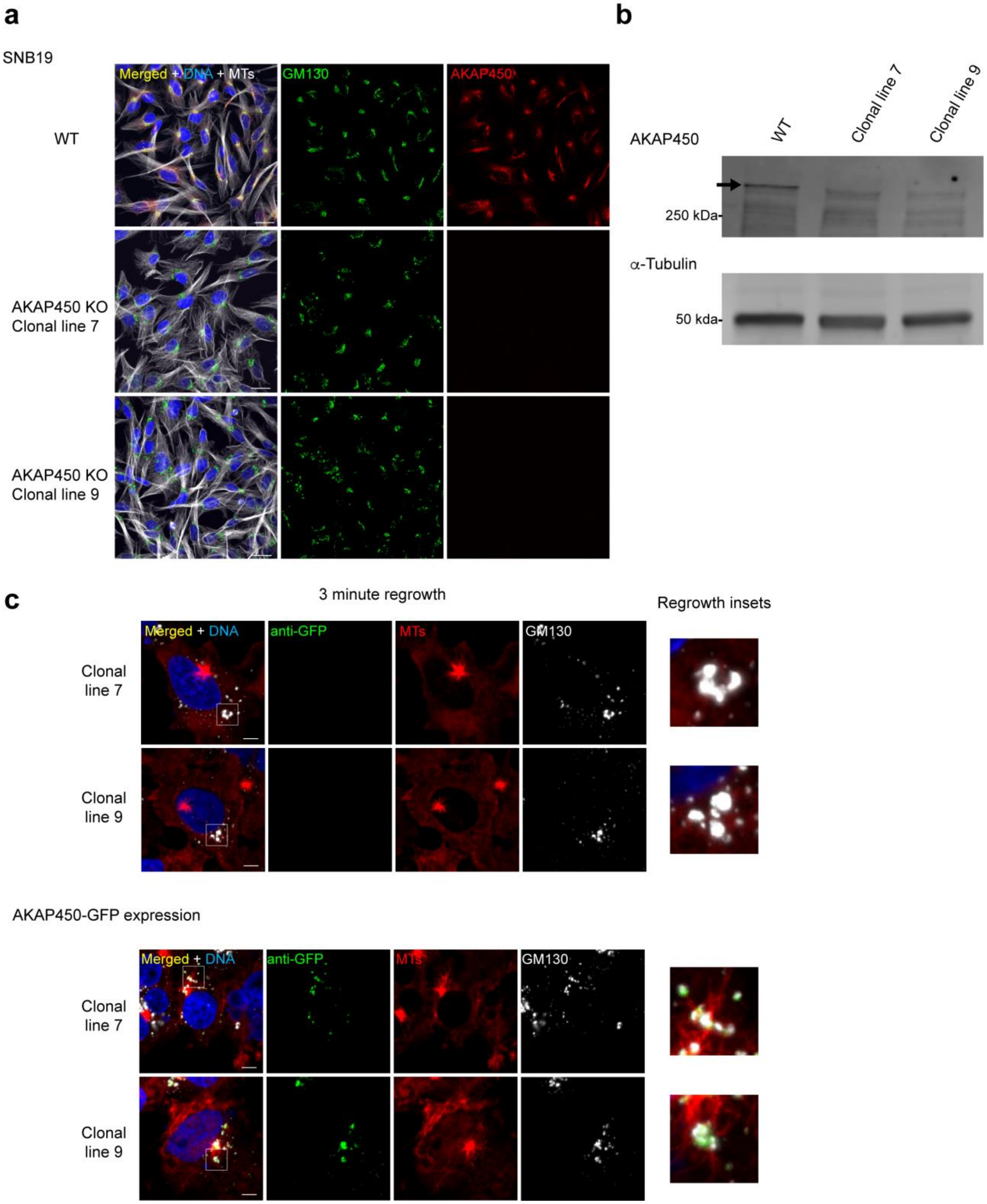

Figure S4: Evaluation of AKAP450 (gene: *AKAP9*) KO SNB19 cells
(**a**)**:** IF staining for GM130 (green), AKAP450 (red), and MTs (white) in WT and AKAP450 KO SNB19 clonal lines 7 and 9.
(**b**): Western blot of AKAP450 from WT and AKAP450 KO clonal lines 7 and 9. An arrow points to the AKAP450 band in WT cells. The loading control is alpha (α)-tubulin.
(**c**): MT regrowth for 3 minutes in AKAP450 KO clones 7 and 9 with and without the expression of AKAP450-GFP. IF staining for anti-GFP (green), MTs (red), and GM130 (white). Zoomed insets show no regrowth from KO cells or regrowth from rescued KO cells from the Golgi.
For all IF staining, DAPI labels the nucleus (blue). Scale bars: 25 μm in (**a**); 5 μm (**c**).

Movie S1: 3D view of the ZIKV mature toroidal viroplasm
3D projection of a ZIKV (MR766) infected SNB19 cell of the ER marked by calnexin (red) staining and the nucleus (blue) labeled through DAPI. Scale bar: 5 μm.

Movie S2: Live imaging of EB3-mApple in WT mock-infected SNB19 cells
Imaging of EB3-mApple (white) comets growing from the centrosome and the Golgi (no marker). The cell was imaged for 2 minutes with 4 seconds per frame. Scale bar: 5 μm.

Movie S3: Live imaging of EB3-mApple in WT ZIKV (MR766)-infected SNB19 cell imaged 24 hours p.i. (Representative #1)
Imaging of EB3-mApple (white) comets growing from the MT cage from the middle section of the cell. The cell was imaged for 2 minutes with 4 seconds per frame. Scale bar: 5 μm.

Movie S4: Live imaging of EB3-mApple in WT ZIKV (MR766)-infected SNB19 cell imaged 24 hours p.i. (Representative #2)
Imaging of EB3-mApple (white) comets growing from the MT cage from the top section of the cell. The centrosome is present but too deep in the cell to see comets. The cell was imaged for 2 minutes with 4 seconds per frame. Scale bar: 5 μm.

Movie S5: Live imaging of EB3-mApple in centrinone-treated AKAP450 KO mock-infected SNB19 cells imaged 24 hours p.i.
Imaging of EB3-mApple (white) comets growing randomly throughout the cytoplasm. The cell was imaged for 2 minutes with 4 seconds per frame. Scale bar: 5 μm.

Movie S6: Live imaging of EB3-mApple in centrinone-treated AKAP450 KO ZIKV (MR766)-infected SNB19 cells imaged 24 hours p.i.
Imaging of EB3-mApple (white) comets growing from the MT cage. The cell was imaged for 2 minutes with 4 seconds per frame. Scale bar: 5 μm.
